# Granulocyte-to-dendritic cell-ratio as marker for the immune monitoring in patients with renal cell carcinoma

**DOI:** 10.1186/2001-1326-3-13

**Published:** 2014-06-06

**Authors:** Dagmar Riemann, Sabine Hase, Kersten Fischer, Barbara Seliger

**Affiliations:** 1 Institute of Med. Immunology, Martin-Luther-University Halle-Wittenberg, Magdeburger Strasse 2, D-06097 Halle, Germany; 2 Clinic of Urology, Martin-Luther-University Halle-Wittenberg, D-06097 Halle, Germany

**Keywords:** Renal cell carcinoma, Flow cytometry, Immune monitoring, Neutrophil-to-lymphocyte ratio (NLR), Dendritic cell

## Abstract

**Background:**

Neutrophil-to-lymphocyte ratio (NLR) has been shown to be a predictor of patients’ outcome for several types of malignancies.

**Findings:**

Using multicolor flow cytometry we searched for predictive markers to monitor blood immune cells in patients with renal cell carcinoma undergoing surgery of the primary tumor. Due to a high standard deviation, pre-surgery NLR values did not significantly differ between tumor patients and the control group. In contrast, the granulocyte-to-dendritic cell (DC) ratio revealed significant higher values in tumor patients. Whereas NLR values did not differ between patients with early stage tumors and locally advanced tumors, the granulocyte/DC ratio was significantly different in these groups. Additionally, comparison of both ratios before and after tumor resection in the two groups “open surgery” and “laparoscopy” could demonstrate the suitability of granulocyte/DC ratio as a marker for immune monitoring.

**Conclusions:**

Granulocyte/DC ratio may serve as a new putative biomarker for the immune monitoring of tumor patients.

## Background

Renal cell carcinoma (RCC) is the most common malignant tumor of the kidney and accounts for approximately 2-3% of all adult malignancies. More than 50% of all renal cancers diagnosed are at a localized stage, however, metastatic disease develops in 20-30% of patients with localized disease [1]. Novel molecular targeted agents, such as tyrosine kinase inhibitors or mammalian target of rapamycin inhibitors change the treatment outcome for patients with metastatic RCC from palliation to prolongation of life. Accurate prediction of long-term disease-free survival immediately after surgical resection of the renal tumor would be valuable for patient counseling, scheduling follow-up imaging and identifying poor risk group patients who might benefit from enrollment in adjuvant therapy protocols. So far, tumor stage, tumor grade and patient performance status remain the most useful and clinically available predictors of the outcome of RCC patients [2]. There exist only few data on prognosis-relevant markers of blood immune cells. A high number of granulocytes has been associated with poorer prognosis in various cancers including RCC [3] and neutrophil count was incorporated into the Heng’s risk classification for metastatic RCC [4].

In our study blood immune cells were investigated in RCC patients undergoing open or laparoscopic tumor surgery. Using multicolor flow cytometry, we focused on the neutrophil-to-lymphocyte ratio (NLR), which has been shown to be a marker of systemic inflammatory response and a predictor of patients’ outcome in this disease [5,6]. We extended our study by the estimation of the granulocyte-to-dendritic cell (DC) ratio as a new putative marker. We compared patients’ data with an age-matched control group, correlated pre-surgery markers with tumor stages and performed time kinetics in patient groups undergoing open or laparoscopic tumor surgery. Our data suggest that the granulocyte/DC ratio is a more valuable marker for the immune monitoring of RCC patients than the NLR.

## Methods

### Patient samples

The study comprised 44 RCC (38 clear cell carcinoma) patients that underwent surgery of the primary tumor at the Department of Urology of the Martin Luther University Halle-Wittenberg. 32 patients had stages < pT3 and 12 patients had locally advanced disease (≥pT3). The average age was 66 years (range 43–86 years). 24 patients were male and 20 were female. 27 patients were operated by open surgery and 17 patients by laparoscopy. EDTA blood was received from tumor patients one day before operation as well as on day 1, 3 and 7 after surgery. The whole blood was analyzed directly, without any cryopreservation or isolation of mononuclear cells. 20 individuals without tumor disease in clinical history and with a mean age of 59 years (range 40–78 years) served as controls. The study was performed with the approval from the Ethics Committee of the Medical Faculty of the Martin Luther University. Written informed consent was obtained from the patients for the publication of this report and any accompanying images.

### Antibodies and Sample Preparation

**Figure 1:**
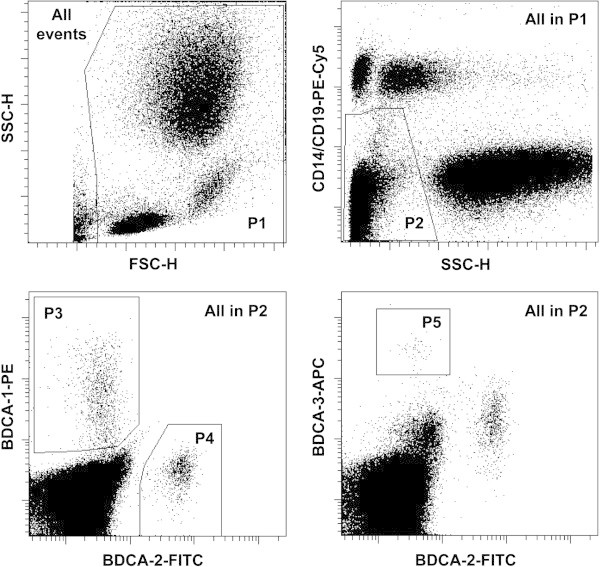
**Gating strategy for the enumeration of blood DC.** Top left: In a forward scatter (FSC)/side scatter (SSC) dot plot with all measured cells a region P1 is drawn to exclude debris. Top right: In a SSC/PE-Cy5 dot plot gated on the P1 population a region P2 is drawn to exclude CD19 positive B cells, CD14 positive monocytes and the granulocytes (with high SSC properties). Down left: In a FL-1/FL-2 dot plot with the cells of population ‘P1 and P2’ a region is drawn around the BDCA-1-PE positive MDC1 cells (P3). Additionally, a region P4 defines the BDCA-2-FITC positive PDCs. Down right: With the cells of population ‘P1 and P2’ a dot plot FL-1/FL-4 is created to define a region with the BDCA-3 strongly positive MDC2s (P5). An isotype control staining involved in the kit helps to set regions in an optimal way. The sum of all blood DC was defined as ‘P1 and P2 and (P3 or P4 or P5)’.

Whole blood cell counts were measured with a Cell-Dyn 3200 (Abbott Laboratories, Wiesbaden, Germany). For flow cytometric analysis, a lysed whole blood technique with 4-color staining of blood cells was used. Lymphocytes and granulocytes were identified on the basis of their forward scatter (FSC) and side scatter (SSC) patterns to get percentages of leukocytes. The staining for CD14 FITC (Beckmann Coulter, Krefeld, Germany) was used to identify monocytes on a FL-1/SSC dot plot. Circulating DC populations were identified with a “Blood DC Enumeration Kit” according to manufacturer’s instructions (Miltenyi, Bergisch Gladbach, Germany). Briefly, aliquots of whole blood were labeled with a cocktail of monoclonal antibodies (mAbs) including anti-CD14-phycoerythrine (PE)-Cy5 and anti-CD19-PE-Cy5 for a dump channel, anti-CD1c-PE as a marker for type-1 myeloid DC (MDC1), CD141/BDCA-3-APC (for MDC2) and CD303/BDCA-2-FITC for plasmacytoid DC (PDC) [7]. After antibody incubation, red cell lysis and washing steps, cells were fixed. Flow cytometric data was acquired on a FACS Calibur cytometer using CellQuest^™^ (BD Biosciences, Heidelberg, Germany). On average, 1x10E6 leukocytes were measured. Figure 1 gives a short overview on the gating strategy for DC subtypes. Results are expressed as percentages of DC in white blood cells or as absolute numbers/μl of blood.

Absolute numbers of granulocytes and lymphocytes were used to estimate the quotient NLR, absolute numbers of granulocytes and DC (as sum of MDC1, MDC2, and PDC) for granulocyte/DC ratio.

### Statistical analysis

Results are given as mean ± SE. Student’s *t*-test, Wilcoxon-Mann–Whitney test, Kruskal-Wallis test and chi-squared test were used to test for differences between groups. All p-values are exploratory.

## Findings

### Comparison of immune cells in RCC patients and a control group

**Figure 2:**
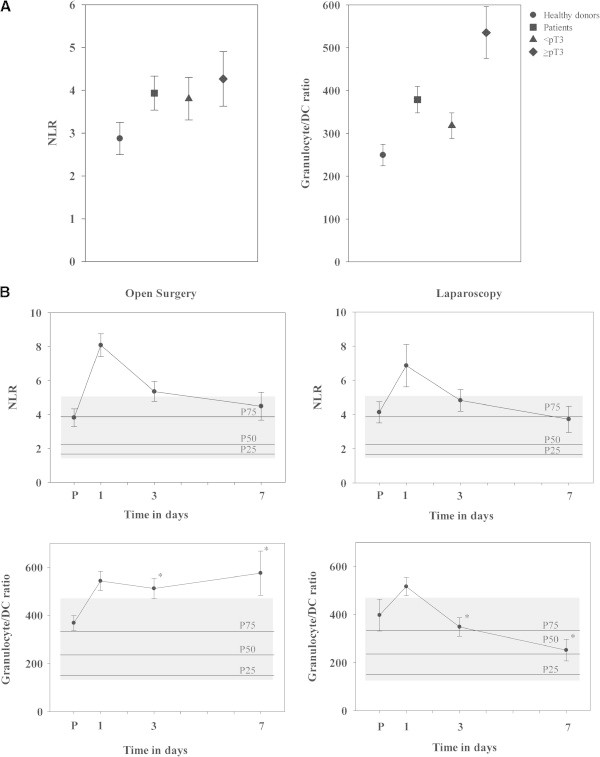
**Comparison of NLR and granulocyte/DC ratio.** NLR and granulocyte/DC ratio were compared between the control group and RCC patients (all patients together, or with patients < pT3 and ≥ pT3), shown in **(A)**. Comparison of both markers in RCC patients undergoing open surgery or laparoscopy **(B)**. An one-week time course of NLR and granulocyte/DC ratio is shown. Values of control group are illustrated as hatched areas and as percentiles, with the 25th percentile (P25), P50 and P75. Significant differences between adjacent points are marked by an asterisk.

In order to determine differences in the frequency of immune cell subpopulations between RCC patients (before surgery) and an age-matched control group, blood cells were analyzed by 4-color-flow cytometry not only for lymphocytes, granulocytes and monocytes but also for DC subpopulations. As summarized in Table 1 there was no significant difference in the number of leukocytes between tumor patients and healthy donors, but patients exhibited a higher percentage of granulocytes (68.8 ± 1.5 versus 61.4 ± 3.4% of leukocytes) and a lower percentage of lymphocytes (23 ± 1.3 versus 27.8 ± 2.1% of leukocytes). DC in peripheral blood are rare (<1% of leukocytes) and the absolute DC numbers in RCC patients and the control group only marginally varied (Table 1). The frequency of all blood DC of RCC patients was lower than those of control group (0.24 ± 0.02 versus 0.32 ± 0.03% of leukocytes, p = 0.028). In particular the MDC1 subpopulation was depressed in patients (p = 0.017), whereas no obvious difference was found for MDC2 as well as for PDC. Figure 2A illustrates that RCC patients had higher NLR values, though this difference was not significant. In contrast, the granulocyte/DC ratio was significantly higher in RCC patients compared to control group (p = 0.002; Figure 2B).

**Table 1:** Comparison of the pretreatment values of blood immune cells of RCC patients with age-matched healthy volunteers

**Parameter**	**Healthy donors**	**RCC patients**	**Patients < pT3**	**Patients ≥ pT3**
Number of leukocytes	6,260 ± 310	7,005 ± 350	6,500 ± 402	8,340 ± 563
Granulocytes	4,227 ± 310	5,103 ± 292	4,702 ± 354	6,139 ± 385
Monocytes	351 ± 39	375 ± 21	349 ± 22	441 ± 42
Dendritic cells (DC)	19 ± 2	16 ± 1	17 ± 1	13 ± 2
Lymphocytes	1,670 ± 111	1,566 ± 103	1,490 ± 110	1,760 ± 237
Neutrophil-to-lymphocyte ratio (NLR)	2.86 ± 0.37	3.93 ± 0,40	3.8 ± 0.5	4.27 ± 0.64
Granulocyte/DC ratio	249.4 ± 25	378.7 ± 31	318.1 ± 30	535.4 ± 60

Data is given as cells/μl or as a quotient, with mean value ± SE.

### Tumor stage-dependent alterations of the immunophenotype

The preoperative immunophenotype of RCC patients with ‘early’ (<pT3) and advanced (≥pT3) stage tumors were compared to investigate tumor stage-dependent alterations (Table 1). The ≥ pT3 group had higher white blood cell counts due to an increased number of granulocytes. Leukocyte numbers correlated with tumor stages (p = 0.031). The number of monocytes and lymphocytes were higher in the advanced group (though not significantly), whereas DC counts were lower. Patients with tumor stages < pT3 possessed 10.9 cells/μl MDC1 and 5.11 cells/μl PDC, while 7.8 cells/μl MDC1 and 4.51 cells/μl PDC were present in the advanced RCC stages ≥ pT3. Both the absolute number and the frequency of DC (as percentage of leukocytes) correlated with tumor staging (p = 0.009). Despite the tumor progression was associated with higher neutrophil counts, no significant differences were observed comparing the NLR values of patients with < pT3 and ≥ pT3 tumor stages due to the high standard deviation (p = 0.607). However, granulocyte/DC ratio was significantly increased in advanced tumor stages (p = 0.001; Figure 2A).

### Comparison of post-operative changes in immune cells between open surgery and laparoscopy

To investigate the immune competence of both patients’ groups, leukocytic subpopulations were measured before (p) as well as one (d1), three (d3) and seven days (d7) after surgery. Most patients showed post-operatively elevated leukocyte counts caused in particular by a short-time increase of granulocytes, whereas the number of lymphocytes and DC dropped. During the post-operative follow up, leukocytic subpopulations recovered, with a faster normalization upon laparoscopy.Both the preoperative NLR and the granulocyte/DC ratio of patients started at or above the 75th percentile of the control group (Figure 2B). NLR values showed a similar course in both surgery groups, with an increase at the first post-operative day and a slow recovery to values of the pre-operative period within 7 days. One day after surgery, the NLR value was 8.08 ± 0.67 in the open surgery group and 6.86 ± 1.24 in laparoscopy (p = 0.348). At day 7 after surgery both patients groups had again a mean NLR level around the 75th percentile of the control group. The difference in the course of DC counts was the major cause for significant differences in the granulocyte/DC ratio between patients with open surgery and laparoscopy at day 3 (p = 0.007) and day 7 (p = 0.03; Figure 2B). DC counts stayed < the 25th percentile of the control group in open surgery, and increased to 133% of the pre-operation value in the laparosopy group.

## Discussion

Using multicolor flow cytometry, the immunophenotype in the blood of renal cancer patients before and after tumor surgery was analyzed to identify surrogate markers for an immune monitoring. In earlier studies, we investigated activation-associated lymphocytic markers as well as monocytic markers, such as HLA-DR and CD13/aminopeptidase N [8], while this study focused on the frequency of leukocytic subpopulations in blood, in particular on DC count. Pretreatment NLR as well as the granulocyte/DC ratio were used as surrogate markers for the immunophenotype. Comparing < pT3 stage RCC to ≥ pT3 tumor stages, higher leukocyte counts were detected in advanced tumor stages due to higher numbers of neutrophils. This is in line with investigations identifying a high neutrophil count as independent factor for poor prognosis in patients with metastatic RCC receiving IL-2 [3] or anti-VEGF (vascular endothelial growth factor) agents [9]. Tumors are known to both drive myelopoiesis, sometimes leading to a clinically apparent leukocytosis, and to inhibit the differentiation of myeloid cells resulting in the accumulation of immature myeloid cells [10]. Cancer-associated myeloproliferation is not merely a paraneoplastic phenomenon of questionable importance, but leads to the suppression of host immunity and promotion of tumor angiogenesis. As an example, arginase-producing myeloid-derived suppressor cells have been revealed as a granulocytic subpopulation in RCC [11].

A high pretreatment NLR is associated with poor prognosis for various cancers including RCC. Multivariate analysis identified increased NLR as an independent prognostic factor for overall, but neither for cancer-specific, nor for metastasis-free survival [5]. In sunitinib-treated patients pre-treatment NLR has been discussed to be associated with progression-free survival [12], and sunitinib even decreases NLR [13]. Often, a NLR higher than 5 has been considered as a critical value correlating with poor prognosis in tumor patients [14]. In our patient group a mean pretreatment NLR of 3.93 was found, with 8 out of 44 patients reaching NLR values higher than 5. However, NLR values showed a high variability (1.2 to 14.4), which prevented a significant difference between control group and tumor patients. Our results showed that combining the number of granulocytes with DC counts is a better approach to monitor immune depression in RCC patients, generating more pronounced differences than NLR. DC as cells specialized in antigen processing and presentation become potent stimulators of an adaptive immune response after undergoing the critical process of maturation. Blood DC represent only the 0.1–0.5% of leukocytes. Based on their lineage origin, they can be divided into two major subsets, plasmacytoid DC as the major producers of type I interferon and myeloid DC [15]. The blood DC counts decrease not only with increasing age [16], but diminished DC frequency and function was found in tumor disease, also in RCC patients [17,18]. It is noteworthy that the frequency of myeloid DC1 has been found to predict progression-free survival in patients with advanced RCC treated with sunitinib [18]. Our results confirm the suitability of blood DC counts for an immune monitoring, and the granulocyte/DC ratio raises differences between RCC patients and control group, or between different tumor stages.

Distinct peri-operative changes have been described in RCC patients, e.g. a down-regulated intensity of monocytic HLA-DR molecules [8]. Granulocytes increase post-operatively and lymphocyte counts decrease, therefore NLR increases at the first postoperative day with higher values in the open surgery group. DC counts decreased after surgery resulting in a raising granulocyte/DC ratio. This marker stayed at high level in the “open surgery” group for the whole observation period of one week, whereas the marker reversed within short time in the laparoscopy group, an observation, which needs further investigation. Understanding the dynamics of DC release and vascular distribution would provide key insights into the process of immune suppression and reconstitution after tumor surgery.

## Conclusion

Taken together, our study implicates the granulocyte/DC ratio as useful tool for monitoring the immunophenotype in RCC patients, e.g. for identifying poor risk group patients who might benefit from enrollment in adjuvant therapy protocols. Larger trials and the correlation of this marker with clinical data, in particular with the patients’ outcome, will be required in the future.

## Abbreviations

NLR: Neutrophil-to-lymphocyte ratio; DC: Dendritic cell; mAbs: Monoclonal antibodies; MDC: Myeloid dendritic cell; PDC: Plasmacytoid dendritic cell; PE: Phycoerythrine.

## Competing interests

The authors declare that they have no competing interests.

## Authors’ contributions

DR was responsible for conception and design of experiments, for analysis and interpretation of data, as well as for drafting the manuscript. SH carried out the flow cytometric acquisition of data and the analysis of data via SPSS. KF collected clinical data of patients. BS revised the manuscript and discussed data. All authors read and approved the final manuscript.
